# The role of purpose in life in healthy aging: implications for the Semmelweis Study and the Semmelweis-EUniWell Workplace Health Promotion Model Program

**DOI:** 10.1007/s11357-025-01625-6

**Published:** 2025-03-29

**Authors:** Virág Zábó, Andrea Lehoczki, Monika Fekete, Ágnes Szappanos, Péter Varga, Marianna Moizs, Giorgia Giovannetti, Yura Loscalzo, Marco Giannini, M. Cristina Polidori, Beatrix Busse, Miklos Kellermayer, Róza Ádány, György Purebl, Zoltan Ungvari

**Affiliations:** 1 Institute of Preventive Medicine and Public Health, Semmelweis University, Budapest, Hungary; 2 Institute of Behavioral Sciences, Faculty of Medicine, Semmelweis University, Budapest, Hungary; 3 Doctoral College, Health Sciences Division, Semmelweis University, Budapest, Hungary; 4 Department of Vascular and Endovascular Surgery, Heart and Vascular Center, Semmelweis University, Budapest, Hungary; 5 Department of Rheumatology and Clinical Immunology, Semmelweis University, Budapest, Hungary; 6 Department of Economics and Management, University of Florence, Florence, Italy; 7 Department of Health Sciences, School of Psychology, University of Florence, Florence, Italy; 8 Aging Clinical Research, Department II of Internal Medicine and Center for Molecular Medicine Cologne, Faculty of Medicine and University Hospital Cologne, University of Cologne, Cologne, Germany; 9 Cologne Excellence Cluster on Cellular Stress-Responses in Aging-Associated Diseases (CECAD), Faculty of Medicine and University of Cologne, Cologne, Germany; 10 Department of Linguistics, University of Cologne, Cologne, Germany; 11 Department of Biophysics and Radiation Biology, Semmelweis University, Budapest, Hungary; 12 Vascular Cognitive Impairment, Neurodegeneration and Healthy Brain Aging Program, Department of Neurosurgery, University of Oklahoma Health Sciences Center, Oklahoma City, OK USA; 13 Stephenson Cancer Center, University of Oklahoma, Oklahoma City, OK USA; 14 Doctoral College, Health Sciences Division/Institute of Preventive Medicine and Public Health, International Training Program in Geroscience, Semmelweis University, Budapest, Hungary; 15 Oklahoma Center for Geroscience and Healthy Brain Aging, University of Oklahoma Health Sciences Center, Oklahoma City, OK USA; 16 Department of Health Promotion Sciences, College of Public Health, University of Oklahoma Health Sciences Center, Oklahoma City, OK USA; 17 Fodor Center for Prevention and Healthy Aging, Semmelweis University, Budapest, Hungary

**Keywords:** Purpose in life, Aging, Public health, Caring University Model Program, Employees, Health risk assessment, Occupational setting, Health promotion, Semmelweis Study

## Abstract

The global aging population presents significant challenges to public health systems, particularly in countries like Hungary, which faces some of the least favorable health indicators in the European Union. To address these challenges, Purpose in Life (PIL) has emerged as a critical determinant of healthy aging, influencing physical, mental, and social health. Defined as a sense of meaning, direction, and intentionality, PIL promotes resilience, mitigates age-related decline, and fosters well-being. This review explores the theoretical frameworks, mechanisms, and practical implications of PIL in the context of aging. Biologically, PIL regulates stress responses, contributing to reduced disease risk and improved longevity. Psychologically, PIL fosters resilience, self-regulation, and positive emotions, which buffer against mental health challenges and support cognitive health. Socially, PIL strengthens meaningful relationships, promotes prosocial behaviors, and fosters collective purpose, reducing isolation and enhancing social cohesion. These mechanisms interact to create a synergistic effect that supports healthy aging trajectories. The Semmelweis Study, Hungary’s most extensive workplace cohort study, offers a unique opportunity to integrate PIL assessment into its longitudinal design, providing novel insights into how PIL influences aging outcomes. Complementing this research, the Semmelweis-EUniWell Workplace Health Promotion Program translates these insights into actionable interventions, designed to enhance employee well-being and productivity. Drawing from global best practices, including insights from Blue Zones and Mediterranean-inspired interventions, Hungary can position PIL as a cornerstone of its healthy aging agenda. Incorporating PIL-focused strategies into workplace health programs and national public health policies holds the potential to extend healthspan, reduce healthcare costs, and foster a resilient and purposeful aging population. This review highlights the transformative potential of PIL in addressing the multifaceted challenges of aging and advancing public health goals.

## Introduction

The global population is aging at an unprecedented rate, with individuals aged 60 and older projected to double by 2050, reaching over two billion worldwide [1]. In Europe, the proportion of individuals over 65 years is expected to surpass 30% by 2058 [2], posing profound challenges to public health systems, labor markets, and social services. Hungary, in particular, faces a rapidly aging and declining population [3, 4], compounded by some of the worst health indicators in the European Union [5]. Life expectancy in Hungary remains one of the shortest in Europe [4, 5], with significant disparities in healthy life expectancy [5], reflecting an urgent need for interventions that extend “healthspan” alongside lifespan.

Unhealthy aging, characterized by accelerated biological aging, early onset of chronic diseases, and reduced functional capacity, is a growing concern in Hungary [5]. Behavioral risks such as smoking, obesity, and low physical activity contribute to this trajectory, leading to high mortality and morbidity rates [5, 6]. Additionally, social determinants, including lower socio-economic status and unequal access to healthcare, exacerbate the burden of aging in the country [7]. Addressing these challenges requires a multifaceted approach that goes beyond biomedical interventions to consider psychosocial assets, such as purpose in life, as critical determinants of healthy aging. In response to these challenges, Semmelweis University, the leading health sciences university in the region, has launched a dual initiative to combat unhealthy aging in Hungary [8, 9]. The Semmelweis Study [9], a longitudinal workplace cohort study involving all university employees, is designed to identify determinants of unhealthy aging, ranging from biological and psychological factors to lifestyle and environmental influences. Complementing this research arm, the Semmelweis-EUniWell Workplace Health Promotion Program aims to translate these findings into actionable interventions that improve the health and well-being of employees.

These initiatives represent a long-term commitment to understanding and addressing the drivers of unhealthy aging. By integrating epidemiological research with targeted health promotion efforts, the Semmelweis initiatives provide a unique platform for developing and testing strategies that can be scaled nationally and internationally. This manuscript focuses on one such promising factor—purpose in life (PIL)—as a key determinant of healthy aging [10–29] and a potential target for workplace-based interventions. PIL, defined as a sense of direction and meaning that guides life decisions and behaviors [30, 31], has emerged as a critical factor in promoting resilience and well-being, particularly in aging populations. Evidence from “Blue Zones,” regions with exceptional longevity and health, underscores the importance of PIL as a driver of healthier and more fulfilling lives [32–34].

Despite the growing recognition of PIL’s benefits, it remains underexplored from many aspects. This review aims to bridge this gap by defining and discussing the theoretical frameworks underlying PIL, exploring methods for measuring and integrating PIL into the Semmelweis Study, identifying workplace interventions that can promote PIL among employees, and offering policy recommendations to enhance the scalability of these approaches. By drawing lessons from global best practices and leveraging the resources of the Semmelweis initiatives, this manuscript seeks to advance the understanding of how PIL can be harnessed to promote healthy aging and beyond.

## Learning from Blue Zones and the Mediterranean experience

The concept of PIL as a cornerstone of healthy aging finds robust support in the practices and lifestyles of regions known as Blue Zones—geographical areas where populations experience exceptional longevity and health. These regions, including Okinawa in Japan, Sardinia in Italy, Nicoya in Costa Rica, Ikaria in Greece, and Loma Linda in California, share commonalities that contribute to their inhabitants’ remarkable aging outcomes [32, 35–38]. Among these shared traits, a strong sense of purpose—referred to as “ikigai” in Okinawa [33, 34] and “plan de vida” in Nicoya [32]—stands out as a critical determinant of both physical and mental well-being. In Blue Zones, PIL is not an abstract concept but a deeply ingrained cultural practice. For example, “ikigai,” often translated as “reason for being,” reflects a life philosophy that encourages individuals to pursue meaningful activities and maintain a positive outlook [33, 34]. Similarly, in Nicoya, “plan de vida” underscores the importance of family, community engagement, and maintaining a sense of contribution to the collective good [32]. These practices guide life decisions, foster resilience and social cohesion, and maintain hope during difficulties and motivation for healthy behaviors. The integration of PIL into daily life is associated with lower rates of chronic diseases [39], enhanced mental health [40], and extended lifespan [41, 42], demonstrating its profound impact on aging trajectories.

Beyond the Blue Zones, the Mediterranean lifestyle provides another compelling model for promoting healthy aging [43–53]. The Mediterranean region, home to Semmelweis University’s EUniWell partners such as the University of Florence and the University of Murcia, exemplifies how diet, social connectedness, and PIL interweave to support healthspan. The Mediterranean diet, characterized by high consumption of vegetables, fruits, nuts, whole grains, and olive oil, is widely recognized for its protective effects against cardiovascular diseases [54], cancer [53], and metabolic disorders [55]. However, the benefits of this lifestyle extend beyond nutrition [43, 44, 46, 47]. Communal eating, intergenerational living, and a culture of leisure and reflection collectively nurture a strong sense of purpose and belonging, reinforcing the psychosocial dimensions of healthy aging.

The lessons from Blue Zones and the Mediterranean experience offer valuable insights for the development of a healthy aging agenda. Both highlight the critical interplay between individual behaviors, cultural norms, and community structures in fostering PIL. Importantly, these practices are not only about promoting health but also about creating environments where purpose thrives. For example, Sardinia’s emphasis on intergenerational respect and caregiving fosters a sense of responsibility and connection. At the same time, Ikaria’s laid-back lifestyle encourages mindfulness and gratitude [38]—both of which align closely with the principles of PIL. A complex experience of connectedness (to nature, to others, to spiritual entities), dedication to spiritual practices, and strong mutual support for coping with difficulties are also frequent in the Blue Zone cultures.

Drawing inspiration from these models, the Semmelweis initiatives aim to integrate culturally relevant practices that promote PIL into their strategies. The Semmelweis Study [9] and the Semmelweis-EUniWell Workplace Health Promotion Program, seamlessly integrated within Semmelweis University’s “Family-friendly University” framework [56], are uniquely poised to adapt these globally inspired insights to Hungary’s distinct socio-cultural context. For instance, the emphasis on community-building activities in the workplace mirrors the social cohesion found in Blue Zone communities. Similarly, promoting mindfulness, reflection, mutual support, solidarity, and opportunities for meaningful engagement can align with the Hungarian cultural emphasis on resilience and family values. By leveraging partnerships with Mediterranean institutions through the EUniWell framework, Semmelweis University can draw on shared expertise to develop interventions that incorporate the best of both worlds: the structured health promotion strategies of academic institutions and the holistic, lifestyle-oriented approaches of Blue Zones and the Mediterranean. This cross-cultural collaboration has the potential to inform scalable models for healthy aging, rooted in purpose, which can be implemented not only in Hungary but also across Europe and beyond. The Semmelweis initiatives seek to adapt these global lessons into a framework that resonates locally, recognizing that the transformative potential of PIL lies not just in individual behaviors but in cultivating environments where purpose is nurtured. By grounding its programs in these proven practices, the university reinforces its commitment to addressing unhealthy aging through innovative, evidence-based, and culturally sensitive approaches. Incorporating the core functions of Blue Zones into well-designed and disseminated workplace-based healthy aging programs could represent a radical new approach to supporting healthy aging.

## Purpose in life: concept and measurement

PIL refers to a sense of direction, meaning, and intentionality in an individual’s life [57]. It encompasses the drive to achieve personally meaningful goals and the belief that one’s actions contribute to a greater good. As a core component of psychological well-being [58], PIL plays a crucial role in resilience, promoting mental, physical, and social health. As PIL serves as a general determinant of healthy psychological functioning, it is also increasingly recognized as a determinant of healthy aging, particularly in its ability to mitigate the adverse effects of stress [58], enhance coping mechanisms [39], foster positive health behaviors, and help avoiding pychologcial problems and mental disorders [39, 59].

### Theoretical frameworks

Several key theoretical frameworks provide insights into the role of PIL in human well-being and aging:

- Logotherapy [31]: Central to logotherapy is the “will to meaning,” the innate drive to find purpose even in the face of adversity. This framework underscores PIL as a fundamental human motivator, critical for psychological resilience and adaptive coping, particularly in later life.
- Socioemotional selectivity theory (SST [60]: SST posits that as individuals perceive time as limited with age, they prioritize emotionally meaningful goals and relationships. PIL becomes a critical focus, fostering emotional well-being and social engagement in older adults.
- Self-determination theory (SDT) [61]: SDT emphasizes intrinsic motivation and the fulfillment of basic psychological needs—autonomy, competence, and relatedness. PIL aligns closely with these needs, promoting engagement in health-enhancing behaviors and overall life satisfaction.

These frameworks collectively highlight how PIL serves as a psychological anchor, contributing to healthier trajectories in aging populations.

### Mechanisms underlying PIL’s role in healthy aging

PIL exerts its positive impact on healthy aging through interconnected biological, psychological, and social mechanisms, each contributing to enhanced resilience and the mitigation of age-related decline.

#### Biological mechanisms

At the biological level, PIL plays a pivotal role in regulating the hypothalamic–pituitary–adrenal (HPA) axis, the body’s primary system for managing stress [62]. Individuals with a strong sense of purpose demonstrate more adaptive stress responses, including lower cortisol levels and faster recovery from stress [30, 63]. These regulatory effects reduce systemic inflammation [64, 65] and oxidative stress [66], both of which are closely linked to chronic diseases and aging-related health deterioration [67].

In addition to its effects on stress regulation, PIL is associated with reduced levels of pro-inflammatory cytokines, such as IL-6 [68]. These cytokines are markers of inflammation that contribute to the pathogenesis of conditions like cardiovascular disease and diabetes [67]. Lower inflammatory activity in purpose-driven individuals supports better metabolic health and longevity [69]. Furthermore, PIL encourages health-promoting behaviors [70], such as regular physical activity [71] and balanced nutrition [72], which activate the body’s antioxidant defenses and support cellular repair mechanisms. Together, these biological effects slow biological aging processes and improve overall health outcomes.

#### Psychological mechanisms

Psychologically, PIL is a cornerstone for resilience, helping individuals recover from adversity and adapt to challenges more effectively. This resilience acts as a protective buffer against mental health conditions that commonly accompany aging, such as depression [73] and anxiety [74]. By fostering a sense of control and direction, PIL enhances self-regulation, encouraging individuals to make healthier lifestyle choices [72, 75], adhere to medical advice [70], and maintain behaviors that support their physical and mental well-being [72]. A strong sense of purpose is also linked to higher levels of positive emotions, including contentment and satisfaction [76]. These emotions have been shown to improve immune function and reduce inflammation [77], reinforcing the health benefits of PIL. Moreover, positive emotions associated with purpose contribute to better cognitive engagement (see, for example, Broaden-and-Build Theory [78]) and psychological stability [79], critical factors for healthy aging.

#### Social mechanisms

The social dimensions of PIL further amplify its benefits for healthy aging [80]. Purposeful individuals are more likely to cultivate meaningful relationships and engage in social interactions that provide emotional support and stability [81]. These robust social networks mitigate stress, reduce feelings of isolation, and enhance mental health [82], particularly in older adults. PIL also drives prosocial behaviors, such as volunteering, mentorship, and other acts of altruism [83]. These behaviors create a sense of connectedness and shared purpose, fostering a supportive community environment. In workplace settings, the shared sense of purpose can strengthen team cohesion and create a culture of mutual support and collaboration, enhancing both individual well-being and organizational productivity.

Through its multifaceted influence on biological, psychological, and social pathways, PIL emerges as a vital determinant of healthy aging. By reducing stress, fostering resilience, and strengthening social connections, PIL mitigates the adverse effects of aging and promotes a higher quality of life across the lifespan [39]. These mechanisms underscore the transformative potential of PIL-focused interventions in fostering health and resilience in aging populations.

### Assessment of PIL

Accurately assessing PIL is essential for understanding its role in healthy aging. Various tools have been developed to measure PIL, often as part of broader psychological well-being assessments:

- Ryff’s Psychological Well-Being Scales [84]: This widely used instrument includes a subscale to assess PIL, measuring individuals’ sense of meaning, goal-directedness, and intentionality.
- Life Engagement Test (LET) [85]: Focused on the extent to which individuals are engaged in personally meaningful activities, LET offers a concise measure of PIL.
- Purpose in Life Test (PIL Test) [86]: A comprehensive scale developed by Crumbaugh and Maholick to measure PIL based on Frankl’s logotherapy framework.

Despite these tools, PIL remains underexplored in the workplace-community context, particularly within aging-related research. A significant gap in the Semmelweis Study is the absence of direct PIL assessment. While the study collects extensive data on mental health and life satisfaction, it does not specifically capture the dimensions of purpose or meaning in life. To address this, subsequent phases of the Semmelweis Study should consider integrating PIL-specific measures, such as adding validated instruments like Ryff’s PIL subscale [84] or the PIL Test [86] to the mental health module. At the same time, Semmelweis Study collects a number of measures indicative of PIL. Incorporating qualitative approaches, such as open-ended questions or narrative interviews, to capture the subjective dimensions of PIL is also an attractive option. Finally, the study design allows for leveraging digital tools and surveys to enable longitudinal tracking of changes in PIL over time and its impact on health outcomes. By integrating these tools, the Semmelweis Study could provide novel insights into the role of PIL in workplace health and aging trajectories.

## Purpose in life: a focus on promoting healthy aging

A growing body of evidence highlights the transformative role of PIL in promoting health and resilience across the aging spectrum. By offering individuals a sense of direction, meaning, and intentionality, PIL serves as a powerful protective factor against the physical, cognitive, and emotional challenges of aging. Research consistently underscores its profound impact on key health outcomes.

### Reduced age-related chronic diseases

One of the most striking findings is the association between a strong sense of purpose and reduced risk of chronic diseases [39]. Studies demonstrate that individuals with higher levels of PIL are significantly less likely to develop cardiovascular diseases [87], prediabetes, and type 2 diabetes [88]. This protective effect is partly attributed to the role of PIL in encouraging healthier lifestyle choices, such as regular physical activity [59, 89], balanced nutrition [90], and adherence to medical advice [39]. Beyond behavior, PIL appears to exert direct biological effects by reducing systemic inflammation [58, 65]—a major contributor to chronic diseases. For instance, lower levels of pro-inflammatory markers like C-reactive protein have been observed in individuals with greater PIL [91], suggesting a link between purpose and improved metabolic health. These findings highlight PIL as a potential target for reducing the burden of chronic illnesses in aging populations.

### Enhanced cognitive function

Cognitive health, a critical component of healthy aging, is also positively influenced by PIL [18, 25–27]. Purpose-driven individuals exhibit better memory performance, verbal fluency, and overall cognitive resilience [92]. Notably, PIL has been shown to buffer against the harmful effects of Alzheimer’s disease pathology, including amyloid plaques and neurofibrillary tangles [11, 93]. In longitudinal studies, older adults with a strong sense of purpose maintained superior cognitive function and were less likely to experience significant cognitive decline [93]. These findings suggest that PIL not only preserves mental acuity but may also delay the onset of dementia-related symptoms, offering a promising avenue for interventions aimed at protecting brain health in aging populations.

### Mental health benefits

The psychological benefits of PIL are equally compelling. A stronger sense of purpose in life significantly enhances meaning-making coping mechanisms by helping individuals reconcile discrepancies between their global beliefs and situational stressors [94]. This process of meaning-making reduces psychological distress and facilitates better adjustment, resilience, and personal growth, particularly in the face of serious illnesses or aging [94, 95]. Research shows that individuals with a strong sense of meaning/purpose in life increases their resilience to depression [73] and anxiety [74], two of the most common mental health issues in later life [1]. For older adults, a well-defined sense of purpose fosters adaptive coping strategies, stress-related growth, and a redefinition of goals or beliefs to align with life changes associated with aging [96], further strengthening the protective effects of PIL against age-related key risk factors like loneliness and social isolation [97].

### Reduced mortality risk

One of the most compelling outcomes linked to PIL is its association with reduced mortality risk. Across diverse populations, studies consistently find that individuals with a strong sense of purpose live longer, independent of socio-demographic factors such as age, gender, and socio-economic status [42]. The mechanisms underlying this relationship include healthier lifestyles [41, 59, 89], better stress regulation [29, 39, 98], and improved physiological markers, such as lower inflammation [58, 64] and more adaptive cardiovascular responses [87]. By fostering a proactive approach to health and well-being, PIL emerges as a crucial determinant of longevity.

### Purpose in life in the workplace: mitigating burnout and enhancing productivity

In workplace settings, PIL has emerged as a critical factor in mitigating burnout—a pervasive issue among employees, particularly in high-stress environments. By providing individuals with a sense of meaning and alignment with their work, PIL fosters engagement [99], resilience [94], and job satisfaction [100] which goes hand in hand with emotional stability at workplaces [101]. Employees who derive purpose from their roles are more likely to experience not only less stress [102] but also greater motivation [99], leading to improved productivity and well-being [102]. Beyond individual benefits, fostering PIL in workplace environments enhances team cohesion and organizational culture [103], creating a supportive ecosystem where employees thrive.

Therefore, incorporating PIL-focused interventions into workplace health promotion programs could yield significant benefits. Strategies such as purpose workshops, mentorship opportunities, and community-building activities can nurture a sense of direction and fulfillment among employees. These interventions improve individual well-being and contribute to organizational success by fostering a healthier, more engaged workforce.

## Semmelweis Study: integrating PIL

The Semmelweis Study [9], launched in 2024, stands as the largest longitudinal workplace cohort study in Central Europe, involving the entire workforce of Semmelweis University. Its ambitious goal is to uncover the determinants of healthy and unhealthy aging, addressing a critical need in aging population of Europe. The study employs a comprehensive methodological approach, integrating data from self-reported surveys, clinical examinations, physiological assessments, and biomarker analyses. This multidisciplinary framework enables the investigation of diverse factors influencing aging, including socio-economic status, lifestyle behaviors, psychosocial determinants, and biological markers. By collecting extensive baseline and follow-up data, the Semmelweis Study offers a robust platform for understanding the complex interactions that shape aging trajectories.

Despite its wide-ranging scope, the study currently lacks a direct assessment of PIL, a critical psychosocial factor with established links to healthy aging. Existing surveys capture information on general well-being, emotional health, and quality of life, yet they do not delve into the dimensions of meaning, intentionality, or goal-directed living. This gap represents a significant opportunity for future exploration, as PIL has been shown to influence key aging outcomes, including resilience, disease prevention, and cognitive health.

To address this gap, subsequent phases of the Semmelweis Study aim to incorporate measures of PIL into its framework. This would involve integrating validated tools, such as Ryff’s Purpose in Life subscale [84], the Life Engagement Test (LET)^85^, or the Purpose in Life Test (PIL Test) [86], into the survey module. These tools would enable the quantification of PIL and its relationship with other psychosocial and biological determinants of aging. Additionally, qualitative methods such as narrative interviews could provide deeper insights into the subjective and cultural dimensions of PIL, offering a nuanced understanding of how purpose manifests in Hungarian populations.

The expanded focus on PIL would also include biological assessments to explore its physiological underpinnings. Biomarkers associated with stress regulation, such as cortisol levels, and inflammatory markers like C-reactive protein, could be examined alongside indicators of biological aging, such as telomere length. These assessments would shed light on the potential biological pathways through which PIL influences health. Furthermore, epigenetic studies could reveal how PIL impacts gene expression related to stress resilience and overall well-being.

Longitudinal studies would be central in evaluating PIL’s impact over time. Tracking changes in PIL across follow-up periods would help elucidate its relationship with critical health outcomes, including cognitive decline, cardiovascular health, and mental well-being. These studies would also allow researchers to assess whether interventions aimed at enhancing PIL lead to measurable improvements in healthspan and reductions in morbidity. The Semmelweis Study could provide compelling evidence for its role as a cornerstone of healthy aging by linking PIL to both behavioral and biological outcomes.

Integrating PIL into the Semmelweis Study offers the potential to generate transformative insights. PIL may act as a mediator between socio-economic factors, such as education and income, and health outcomes by influencing stress resilience [29, 39, 98], coping mechanisms [94], and motivation for health-promoting behaviors [70].

Furthermore, understanding how PIL interacts with biological pathways, including inflammation and stress regulation, could uncover novel intervention targets for promoting resilience and reducing disease risk. Examining PIL within the Hungarian workplace context may also reveal unique cultural drivers of purpose, enabling the development of tailored strategies to enhance well-being and support healthy aging. Through these advances, the Semmelweis Study would expand the scientific understanding of PIL and pave the way for its integration into public health policies and workplace interventions, offering a holistic approach to addressing Hungary’s aging challenges.

### Cultural adaptation of PIL-related lifestyle factors in Europe

Understanding and integrating PIL into the Hungarian context requires careful consideration of the country’s unique cultural, historical, and socio-economic background. While PIL is universally recognized as a critical determinant of healthy aging, its expression and relevance may vary across cultures, influenced by societal values, traditions, and lived experiences. In Europe, a region shaped by cultural diversity and a complex history and transitioning socio-economic structures, PIL may be deeply intertwined with resilience, familial bonds, and community engagement. For many Europeans, the sense of purpose is often rooted in relationships with family and the cultural emphasis on perseverance and hard work. These factors create an opportunity to explore PIL through a lens that resonates with local values while drawing inspiration from global best practices, such as those found in the Blue Zones [32–34, 98] and Mediterranean regions [54, 55].

To adapt PIL assessments and interventions, the Semmelweis Study must employ culturally sensitive approaches. This begins with the development or adaptation of validated tools that capture not only the universal dimensions of PIL but also its unique manifestations in European societies. For example, incorporating narrative interviews or qualitative methods could provide insights into how purpose is defined and sustained in different European contexts. These approaches would allow participants to articulate their sources of meaning, whether derived from personal achievements, community contributions, or family connections.

Moreover, interventions promoting PIL must align with the local cultural norms and address potential barriers. For instance, fostering community-oriented activities that reflect Europe’s strong tradition of social gatherings and shared responsibilities could enhance the relevance of PIL-focused programs. Similarly, integrating workplace strategies, such as mentorship initiatives and collective goal-setting exercises, into the Semmelweis-EUniWell Workplace Health Promotion Program could help reinforce purpose within professional settings.

The “Family-friendly University” framework [56] at Semmelweis University provides a natural foundation for these efforts, emphasizing the importance of work-life balance, intergenerational collaboration, and personal fulfillment. By positioning PIL within this framework, the university can encourage employees to view their participation in the Semmelweis Study and related programs as a way to enhance their well-being and that of their families and colleagues.

Ultimately, the cultural adaptation of different PIL-related practices in European communities must prioritize a holistic and inclusive approach, ensuring that strategies resonate across different demographic groups and professional roles. By tailoring assessments and interventions to the unique needs and values of Hungarian society, the Semmelweis initiatives can serve as a model for integrating PIL into aging research and health promotion programs across Europe. This culturally attuned framework has the potential to enhance the impact of PIL-focused efforts, fostering healthier, more purposeful lives for Europe’s aging population.

## Semmelweis-EUniWell Workplace Health Promotion Program: targeting PIL

The Semmelweis-EUniWell Workplace Health Promotion Program serves as a flagship initiative within the institution’s broader mission to develop a scalable and impactful framework for promoting healthy aging in workplace settings, both in Hungary and internationally (Ungvari et al. 2025, manuscript in preparation). This program integrates the goals of the European University for Well-Being (EUniWell) framework, emphasizing the collective strengths and best practices of its partnering institutions. EUniWell’s mission of advancing well-being through collaboration and innovation provides a foundation for this initiative, aligning with Semmelweis University’s commitment to creating a supportive, health-conscious environment conducive to fostering resilience and longevity. The program adopts a holistic approach, drawing from evidence-based interventions developed within EUniWell’s network of universities. By leveraging insights from partners such as the University of Florence, University of Murcia, and the University of Cologne, the Semmelweis-EUniWell Workplace Health Promotion Program is uniquely positioned to address the multifaceted dimensions of healthy aging in a workplace setting. This integration of international best practices reflects a shared commitment to enhancing well-being across academic institutions while tailoring solutions to the unique socio-cultural context of Hungary.

### Promoting healthy aging at workplace settings

At the heart of the program is a comprehensive suite of strategies aimed at improving the physical, mental, and social well-being of university employees (Ungvari et al. 2025, manuscript in preparation). Current interventions include programs designed to encourage physical activity, such as workplace exercise initiatives and ergonomic workplace designs. Nutritional guidance is also offered, with an emphasis on promoting balanced diets and healthy eating habits [8]. Counseling services provide critical support for stress management and mental health, addressing the growing burden of workplace stressors [8]. Additionally, resilience training and stress-management workshops help employees develop coping mechanisms and navigate professional and personal challenges with greater ease.

While these initiatives have shown positive outcomes (Fazekas-Pongot et al. 2025, manuscript in preparation), the program recognizes the need to address deeper psychosocial factors, particularly PIL. PIL has emerged as a transformative factor in promoting well-being and resilience, serving as a key driver of health and motivation. A sense of purpose is associated with reduced burnout [104], enhanced engagement [105], and improved overall satisfaction [106], making it a pivotal target for interventions aimed at fostering healthy aging within the workplace.

To address this gap, the Semmelweis-EUniWell Workplace Health Promotion Program is expanding its scope to include interventions specifically designed to cultivate PIL among employees. One proposed strategy is the introduction of purpose workshops, where participants are guided through reflective exercises to identify their life goals, personal values, and sources of meaning. These workshops aim to align employees’ professional roles with their broader sense of purpose, fostering a sense of fulfillment and direction.

Mentorship programs are another proposed initiative, pairing senior employees with younger colleagues to facilitate knowledge transfer and career development. This mentorship model not only benefits mentees by providing guidance and inspiration but also allows mentors to derive purpose and satisfaction from contributing to the growth of future professionals. These relationships have the potential to deepen connections across generations, reinforcing a shared sense of mission within the workplace.

In addition to individual-focused strategies, community-building activities are being implemented to strengthen collective purpose and foster a sense of belonging among employees. Recognizing the value of shared experiences and collective goals, the program prioritizes initiatives such as team-building events, volunteer projects, and collaborative efforts that contribute to societal well-being. These activities are designed to enhance social bonds and create an environment where employees feel connected not only to their colleagues but also to the overarching mission of the university. A key element of this approach is the integration of participation in the Semmelweis Study within the broader framework of community-building. The university leadership actively promotes the idea of a “Semmelweis family” mentality [8], positioning participation in the Semmelweis Study as both a personal and collective commitment to health and well-being. Employees are made aware that the study is not merely a research initiative, but a program designed for them—a tool to identify risk factors and empower them with actionable knowledge. Participants who are identified as having potential health risks through the study are offered the opportunity to consult the Preventive Medicine Practice recently launched by the university, which provides individualized guidance and interventions based on the principles of lifestyle medicine. This integration underscores the university’s dedication to healthy aging by connecting employees to evidence-based resources and support systems. Participation in the Semmelweis Study also serves as a gateway to the broader programs offered by the Semmelweis-EUniWell Workplace Health Promotion Program, ensuring that employees are channeled into workshops, resilience training, and other interventions tailored to their specific needs. This cohesive strategy not only facilitates early intervention but also reinforces the sense of community by demonstrating the university’s investment in its employees’ health and longevity. By aligning individual health goals with collective well-being, the university is building a culture that empowers its workforce, fosters engagement, and instills a shared sense of purpose. Employees are encouraged to see themselves not only as participants in the programs, but as integral members of a supportive and forward-thinking community dedicated to advancing health and resilience. Through this holistic approach, the university leadership aims to cultivate a workplace environment where every individual feels valued, connected, and motivated to contribute to the shared mission of promoting healthy aging in Hungary and beyond.

The success of these interventions will be measured using a robust evaluation framework. Metrics such as employee-reported quality of life, levels of job satisfaction, and engagement will provide insight into the effectiveness of PIL-focused initiatives. Organizational outcomes, including productivity, absenteeism, and staff retention rates, will also be monitored to assess the broader impact of the program. Furthermore, health outcomes such as reductions in stress-related illnesses and improvements in physical and mental health will serve as critical indicators of the program’s contributions to healthy aging.

By explicitly targeting PIL, the Semmelweis-EUniWell Workplace Health Promotion Program goes beyond traditional health interventions to address the deeper psychosocial needs of employees. This approach not only supports individual well-being but also aligns with the EUniWell mission to foster resilience and thriving communities across academic institutions. As the program evolves, it offers a model for workplace health strategies that can be adapted and scaled across Hungary and beyond. Through its focus on purpose and well-being, the program represents a significant step toward creating a healthier, more resilient workforce, advancing the broader goal of healthy aging in Hungary.

### Developing a Mediterranean-inspired framework for healthy aging

Drawing on the rich traditions and proven health benefits of the Mediterranean lifestyle, the development of a Mediterranean-inspired framework for healthy aging offers a compelling pathway to enhance the impact of the Semmelweis-EUniWell Workplace Health Promotion Program. This approach builds on the shared expertise within the EUniWell network, particularly with Mediterranean partners such as the University of Florence and the University of Murcia, to adapt elements of the Mediterranean lifestyle to Hungary’s workplace and cultural context. At the core of this framework is the recognition that the Mediterranean lifestyle encompasses more than just diet; it is a holistic model that integrates physical activity, social cohesion, and a strong sense of purpose. The Mediterranean diet, characterized by its emphasis on plant-based foods, healthy fats, and moderate consumption of fish and wine, is widely celebrated for its protective effects against cardiovascular disease, metabolic disorders, and age-related cognitive decline. However, equally significant are the social and cultural practices associated with the Mediterranean way of life, such as communal meals, intergenerational support systems, and opportunities for leisure and reflection. To integrate these principles, the Semmelweis-EUniWell Workplace Health Promotion Program will implement workplace interventions that emphasize not only healthy eating but also the psychosocial dimensions of well-being. These include culinary workshops and communal meals could foster healthier eating habits while strengthening team bonds. Intergenerational mentorship programs could replicate the Mediterranean tradition of elders sharing wisdom and guidance, fostering purpose and connection across age groups. Leisure activities and mindfulness practices encourage relaxation and promote emotional resilience. The Mediterranean model’s alignment with the concept of PIL further enhances its relevance. Integrating these aspects into the Semmelweis-EUniWell Workplace Health Promotion Program amplifies its impact on employees’ overall well-being and productivity. This Mediterranean-inspired framework for healthy aging also serves as a forward-looking strategy for scaling the program’s initiatives. By leveraging the EUniWell network for collaborative research and pilot programs, Semmelweis University can refine and validate these interventions, creating a model adaptable to diverse workplace and cultural settings across Europe. As Hungary seeks to address its aging challenges, this framework positions the university as a leader in promoting resilience, longevity, and well-being through culturally attuned, evidence-based approaches.

## PIL as a public health imperative in Hungary

By integrating PIL assessment and targeted interventions into the Semmelweis Study and the Semmelweis-EUniWell Workplace Health Promotion Program, it will be possible to develop a National Healthy Aging program domain to address important aging challenges in Hungary in a novel and impactful way. Purpose in life offers a transformative pathway to healthier, more fulfilling lives, particularly for aging populations facing socio-economic and health disparities. Emphasizing PIL as both a research focus and an intervention target represents a critical step toward extending healthspan, improving quality of life, and creating a more resilient society.

The integration of PIL into public health policies represents a transformative opportunity to address the pressing challenges of unhealthy aging in Hungary. PIL, as a psychosocial determinant of health, has been consistently linked to reduced risk of chronic diseases [39, 87–89, 107, 108], enhanced cognitive and mental health [39, 40], and improved quality of life [39]. Its inclusion in public health strategies could yield far-reaching benefits, including substantial reductions in healthcare costs, improved population health outcomes, and increased healthspan.

By fostering a strong sense of purpose, individuals are more likely to adopt healthier behaviors, such as regular physical activity [59, 89], balanced nutrition [90], and adherence to medical recommendations [39]. Furthermore, PIL has been shown to reduce systemic inflammation [64], regulate stress responses [63, 109], and bolster resilience against the onset of chronic conditions such as cardiovascular disease [87, 110], diabetes [88], and depression [111]. These protective effects not only enhance individual well-being but also alleviate the burden on Hungary’s healthcare system, which is currently strained by the high prevalence of age-related diseases.

Scaling findings from the Semmelweis Study and the Workplace Health Promotion Program into national health promotion campaigns could catalyze a broader cultural shift toward healthier, purpose-driven living. National campaigns could emphasize the importance of identifying and nurturing PIL, particularly among older adults and working populations. Messaging could focus on practical steps individuals can take to cultivate a sense of purpose, such as engaging in community service, setting meaningful personal goals, and building stronger social connections.

Moreover, targeted interventions could be rolled out in collaboration with local governments, healthcare providers, and community organizations. For example, integrating PIL-focused workshops into existing public health initiatives, such as workplace wellness programs or senior citizen activities, could extend the reach and impact of these efforts. Hungary’s commitment to promoting healthy aging must recognize PIL not only as a research focus but also as a cornerstone of public health strategy, capable of fostering resilience and well-being at a national scale.

### Policy recommendations for national healthy aging programs

Incorporating PIL into Hungary’s national aging strategy requires actionable policies that address the multifaceted nature of healthy aging. To integrate PIL into existing and new public health frameworks, several strategic recommendations emerge. A critical avenue for implementation is the integration of PIL into workplace health initiatives. Workplaces serve as ideal environments to foster a sense of purpose, given their significant role in daily life and social identity. Policies could recommend the inclusion of PIL assessments and interventions as part of workplace wellness programs across both public and private sectors. These interventions might include purpose workshops, mentorship initiatives, and team-building activities designed to enhance employee well-being and productivity. Furthermore, governments could incentivize employers to implement these programs to encourage broader adoption. Public education campaigns represent another essential strategy for embedding PIL into the national healthy aging agenda. Public education campaigns represent another essential strategy for embedding PIL into the national healthy aging agenda. National awareness campaigns could emphasize the role of PIL in fostering resilience, reducing stress, and enhancing overall health outcomes. Such campaigns should use relatable examples and success stories to inspire individuals to cultivate their own sense of purpose. Community-based programs can further enhance the reach and impact of PIL-focused initiatives. Enhancing and leveraging the existing network of Health Promotion Offices in Hungary could facilitate the implementation of PIL-focused activities, such as intergenerational mentorship programs, volunteer opportunities, and cultural events. These initiatives would not only strengthen social bonds but also offer individuals meaningful avenues for engagement, fostering a greater sense of purpose and community cohesion. Collaboration with religious, cultural, and civic organizations would leverage existing community networks, ensuring that programs are culturally resonant and accessible to diverse populations. Finally, embedding PIL within national policies is crucial for ensuring its sustainability and impact. PIL should be recognized as a key component of Hungary’s National Healthy Aging Strategy, aligned with other public health priorities such as physical activity promotion, mental health support, and chronic disease prevention. The integration of PIL into public health initiatives not only addresses the immediate challenges of an aging population but also builds a resilient and engaged community, ready to adapt to the demands of a rapidly changing demographic landscape. Through these measures, Hungary can lead the way in developing innovative and impactful strategies for promoting healthy aging at the national level.

## Conclusion

Integrating PIL into public health strategies represents a transformative opportunity to combat unhealthy aging. Purpose-driven initiatives can significantly enhance healthspan and reduce the burden of aging-related challenges by fostering resilience, promoting mental and physical well-being, and strengthening social bonds. The Semmelweis Study and Semmelweis-EUniWell Workplace Health Promotion Program provide a robust platform for advancing this agenda, offering scalable models for both national and international applications. To address the complexities of aging, it is essential to prioritize psychosocial assets like PIL alongside biological and lifestyle interventions. By embedding PIL into Hungary’s healthy aging strategies, the nation can create innovative, holistic approaches that empower individuals to live healthier, more purposeful lives. This vision addresses the immediate challenges of aging populations and builds a foundation for a more resilient and engaged society, both in Hungary and beyond.
